# *Efunaviria, Floreoviria, Pleomoviria,* and *Volvereviria*: four realms of viruses with small DNA genomes to replace realm *Monodnaviria*

**DOI:** 10.1128/jvi.01019-26

**Published:** 2026-08-12

**Authors:** Mart Krupovic, Arvind Varsani, Simon Roux, F. Murilo Zerbini, Jens H. Kuhn, Eugene V. Koonin

**Affiliations:** 1 Institut Pasteur, Université Paris Cité, Cell Biology and Virology of Archaea Unit 27058, Paris, France; 2 The Biodesign Center for Fundamental and Applied Microbiomics, Center for Evolution and Medicine, School of Life Sciences, Arizona State University 69991, Tempe, Arizona, USA; 3 DOE Joint Genome Institute, Lawrence Berkeley National Laboratory 1666, Berkeley, California, USA; 4 Departamento de Fitopatologia/BIOAGRO, Universidade Federal de Viçosa 447238, Viçosa, Minas Gerais, Brazil; 5 Independent Researcher 827085 , Frederick, Maryland, USA; 6 Computational Biology Branch, Division of Intramural Research, National Library of Medicine, National Institutes of Health 841480 , Bethesda, Maryland, USA; Dartmouth College Geisel School of Medicine, Hanover, New Hampshire, USA

**Keywords:** virus evolution, virus diversity, megataxonomy, International Committee on Taxonomy of Viruses, capsid proteins, ICTV

## Abstract

Realm *Monodnaviria* was established for unification of viruses with small DNA genomes that encode homologous endonucleases initiating rolling circle replication or their inactivated derivatives. However, comprehensive analysis of the expanded virus sequence and structure databases revealed that neither replication nor structural modules within this realm were monophyletic. Consequently, *Monodnaviria* was reorganized by moving three of its four kingdoms to separate new, likely, monophyletic realms, *Efunaviria*, *Pleomoviria,* and *Volvereviria*, and renaming the remaining realm *Monodnaviria* to *Floreoviria*.

## COMMENTARY

**Fig 1:**
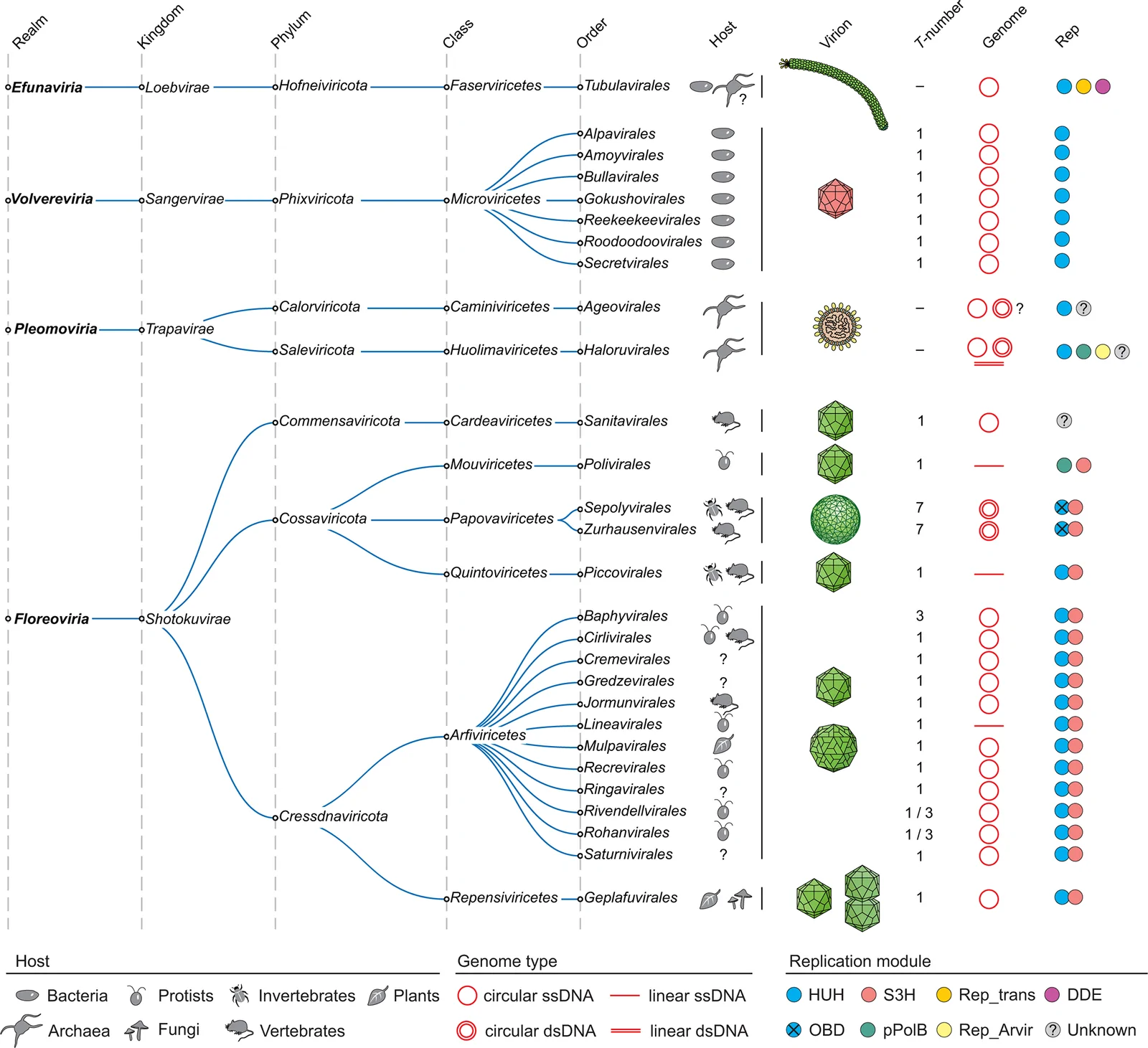
Four realms of viruses with small DNA genomes. The figure shows the current (2026) taxonomic organization of each realm down to the rank of order. Columns on the right depict the virion organization, known hosts, genome type and topology, and replication proteins for each group of viruses. For viruses producing icosahedral capsids, the triangulation (*T*) number is also indicated. Uncertainty is indicated with question marks. Rep, replication protein; HUH, HUH superfamily endonuclease; OBD, origin-binding domain derived from an inactivated HUH endonuclease; S3H, superfamily 3 helicase; pPolB, protein-primed family B DNA polymerase; DDE, DDE superfamily transposase. The data used to create the taxonomic tree were retrieved from the ICTV website (3). Virion diagrams were obtained from ViralZone (4), licensed under a Creative Commons Attribution 4.0 International (CC BY 4.0) License.

Realm *Monodnaviria* was established in 2020 to unify viruses with small single-stranded DNA (ssDNA) or double-stranded DNA (dsDNA) genomes that replicate using homologous rolling circle replication initiation endonucleases (RCREs) of the HUH superfamily (named after the conserved motif including two histidine residues separated by a bulky hydrophobic residue) or their inactivated derivatives (1, 2). The realm included four kingdoms, *Loebvirae*, *Sangervirae*, *Shotokuvirae*, and *Trapavirae*, which comprised bacteria-infecting viruses producing filamentous virions, bacteria-infecting viruses forming small icosahedral capsids, eukaryote-infecting viruses producing icosahedral capsids, and archaea-infecting viruses associated with pleomorphic virions, respectively (Fig. 1).

The realm was created on the premise that viruses from the four kingdoms shared the homologous HUH superfamily RCRE (1). However, the expanded exploration of virus diversity, complemented by recent comparative sequence and structural analyses, showed that replication proteins encoded by viruses from different kingdoms of *Monodnaviria* are not orthologous and, in many cases, not homologous (Fig. 1). Furthermore, the structural modules of these viruses, i.e., the sets of genes encoding proteins involved in virion morphogenesis and structure, are also unrelated. Thus, the monophyly of the realm *Monodnaviria* could not be justified by the conservation of either the structural or the replication module. To address this discrepancy, in 2026, three kingdoms were moved from *Monodnaviria* into newly established, separate realms (*Efunaviria*, *Pleomoviria*, and *Volvereviria*), and the remaining realm *Monodnaviria* was renamed *Floreoviria*.

## REALM *EFUNAVIRIA*, KINGDOM *LOEBVIRAE*

**Fig 2:**
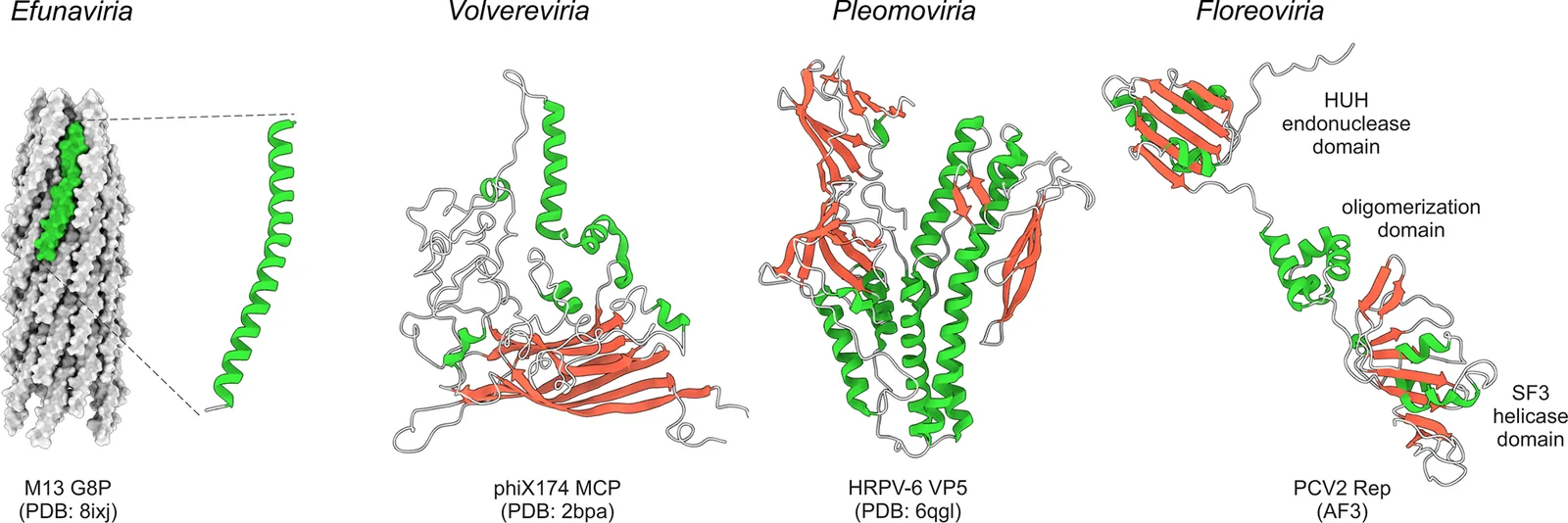
Signature proteins of the realms *Efunaviria*, *Volvereviria*, *Pleomoviria,* and *Floreoviria*. The structural models are colored according to the secondary structure elements: α-helices, green; β-strands, red; random coil, gray. Realm *Efunaviria* is characterized by a unique morphogenesis module, which includes the FtsK ATPase and the hydrophobic α-helical capsid protein (G8P in Escherichia phage M13). Here, only the capsid protein is depicted, both as a single subunit (right) and in the context of the helical virion (left). One subunit is shown in green, whereas other subunits are shown in gray using surface representation. Realm *Volvereviria* is represented by the major capsid protein (MCP) of Escherichia phage phiX174. *Pleomoviria* is represented by the membrane fusion protein VP5 of Halorubrum pleomorphic virus 6 (HRPV-6). The PDB accession numbers are indicated under the corresponding structures. *Floreoviria* is represented by the AlphaFold3 (AF3) structural model of the replication initiation protein (Rep) of porcine circovirus 2 (PCV2; GenBank protein accession AAP44182).

Realm *Efunaviria* includes bacteria-infecting viruses with circular ssDNA genomes that are packed within helically symmetrical, non-enveloped filamentous particles (5–7). The morphogenetic module of filamentous phages and their virion morphogenesis process are unique among viruses. The ssDNA replicative intermediate of the virus genome is extruded through the host cytoplasmic membrane, with the major capsid protein (MCP) subunits, which are transiently embedded within the membrane, assembling around the rod-shaped DNA in a helical fashion (Fig. 2), dissociating from the membrane in the process (7). The tips of the filamentous particles are decorated with minor capsid proteins, including those involved in host recognition and binding (6). An FtsK family ATPase plays a key role in the ATP-driven genome extrusion through the membrane (7).

The realm includes a single order, *Tubulavirales*, with three families, 66 genera, and 110 species. Furthermore, over 10,000 putative efunavirian genome sequences have been identified across bacterial diversity and multiple types of ecosystems by mining genomic and metagenomic data, illustrating the diversity of these viruses and likely representing many new families and/or genera (8). Notably, potential archaea-infecting members of the realm were also predicted (Fig. 1). Analysis of this greatly expanded set of efunavirian genomes revealed that, whereas the morphogenetic module is relatively well conserved across the kingdom, the replication modules vary greatly. Although all efunavirians package ssDNA molecules, this replicative form of the genome can be generated using different mechanisms, catalyzed by non-homologous enzymes (8) (Fig. 1). The rolling circle replication, the dominant genome replication mechanism among efunavirians, is catalyzed by at least two non-homologous, structurally distinct RCREs. The most common RCRE among efunavirians belongs to the Rep_trans family, which is widespread among bacterial plasmids and adopts a distinct TATA-binding protein (TBP)-like structural fold (9). By contrast, the HUH superfamily enzymes encoded by a minority of efunavirians adopt the RNA recognition motif (RRM)-like fold (2, 10). Besides the Rep_trans and HUH superfamily RCREs, some efunavirians encode RepL family replication proteins (Rep) (8), structurally related to helix-turn-helix DNA-binding proteins and widespread in plasmids of gram-positive bacteria (11). Finally, certain viruses of the *Plectroviridae* family lack dedicated genes for the genome replication and instead appear to produce the ssDNA intermediates during transposition catalyzed by IS*3* and IS*30* family transposases of the DDE superfamily (named after the catalytic triad of three amino acid residues, typically, two aspartyls and one glutamyl) (12).

Clustering analysis of the HUH superfamily RCREs encoded by efunavirians showed that they are not monophyletic but rather have been acquired from plasmids on multiple independent occasions (13). Thus, there is no evidence that the ancestor of efunavirians encoded an HUH superfamily RCRE. Given the extent of gene replacement, the replication module does not appear to reflect the evolutionary history of this virus realm. Instead, the morphogenetic module, in particular, the highly hydrophobic α-helical MCP and the FtsK-like ATPase, appears as an appropriate hallmark.

Etymology: *Efunaviria*, after Latin letter “ef” and Latin “una” for “one,” a reference to Enterobacteria phage f1 (*Inoviridae: Inovirus M13*).

## REALM *VOLVEREVIRIA*, KINGDOM *SANGERVIRAE*

Realm *Volvereviria* consists of viruses with small circular ssDNA genomes forming icosahedral capsids. The realm includes the expansive class *Microviricetes*, with seven orders, 21 families, 115 genera, and 131 species, exemplified by the iconic Escherichia phage phiX174 (*Eubullaviridae: Sinsheimervirus phiX174*), whose genome was one of the first DNA genomes to be sequenced (14). Volverevirians are genetically vastly diverse, with many unclassified representatives discovered by metagenome mining across ecosystems (15–18). All isolated volverevirians infect bacteria, but the hosts for the vast majority of viruses in this realm remain unknown. Despite the vast genetic diversity, volverevirians are relatively uniform in terms of both morphogenetic and replication modules (Fig. 1). The morphogenetic module commonly consists of an MCP with a single jelly-roll (SJR) fold and a pilot protein involved in genome delivery into the host cell but may also include scaffolding proteins, spike proteins, and other minor components of the virion (19, 20). Volverevirians appear to uniformly form small *T* = 1 icosahedral capsids. The replication module primarily includes HUH superfamily RCREs. Notably, neither the MCP nor the RCRE of volverevirians are closely related to homologs from other known viruses, including members of the three other former monodnavirian kingdoms, suggesting an independent origin of this virus group. Although either the MCP or RCRE could be, in principle, chosen as a hallmark for classification of volverevirians, it is the MCP that has been historically used for delineating the phylogenetic relationships among viruses within this realm (Fig. 2).

Etymology: *Volvereviria*, after Latin “volvere” for “to roll” in reference to rolling circle replication, uniformly used by viruses in this realm, and jelly-*roll* fold capsid proteins conserved in all volverevirians.

## REALM *PLEOMOVIRIA*, KINGDOM *TRAPAVIRAE*

Realm *Pleomoviria* consists of archaea-infecting viruses forming enveloped pleomorphic virions and currently includes two phyla, *Calorviricota* and *Saleviricota*. Phylum *Saleviricota* includes two families, *Pleolipoviridae* for viruses with linear or circular dsDNA or ssDNA genomes that infect extremely halophilic archaea (21), and *Nanopleoviridae* for divergent relatives of pleolipovirids infecting ultra-small archaea of the phylum *Nanohaloarchaeota* (22). Phylum *Calorviricota* includes a single family, *Thalassapleoviridae*, for viruses infecting deep-sea hyperthermophilic archaea (23) (Fig. 1). Several additional groups of putative pleomovirians infecting methanogenic archaea have been described (24) but remain to be formally classified.

Membership in *Pleomoviria* is based on the morphogenetic module, which consists of an archaea-specific membrane fusion protein and a conserved membrane-embedded matrix protein (25). The matrix proteins are highly divergent at the sequence level, whereas the fusion proteins are sufficiently conserved and are thus conducive to phylogenetic analysis and structural comparisons. By contrast, the replication modules of pleomovirians are particularly diverse (Fig. 1). Alphapleolipoviruses have ssDNA genomes and encode HUH superfamily RCREs, but these are not monophyletic and apparently have been exchanged with plasmids of different families on several independent occasions (13, 21). Most viruses in the genus *Betapleolipovirus* encode an archaea-specific RCRE of the TBP-fold family, Rep-Arvir, which is distinct from the HUH superfamily enzymes and distantly related to the bacterial Rep_trans RCRE (26). Like in the case of HUH RCREs of alphapleolipoviruses, Rep-Arvir enzymes have been exchanged horizontally with other groups of archaeal viruses and plasmids. By contrast, gammapleolipoviruses encode protein-primed family B DNA polymerases and contain linear dsDNA genomes (27). Similarly, in *Thalassapleoviridae*, members of only one of the three genera encode HUH RCREs and appear to replicate by the rolling-circle mechanism (23). Finally, certain members of the *Nanopleoviridae*, *Pleolipoviridae,* and *Thalassapleoviridae* do not encode recognizable replication proteins and most likely rely on distinct, host-encoded replication factors (22, 23, 28). Thus, even relatively closely related pleomovirians, belonging to the same family, encode non-homologous replication proteins, which are rampantly exchanged with evolutionarily unrelated viruses and plasmids, emphasizing the impossibility of using any given replication module as a hallmark of this virus realm. Conversely, the morphogenetic module, especially the fusion protein (Fig. 2), is specific to this virus realm and distinguishes them from all other characterized viruses.

Etymology: *Pleomoviria*, after the pleomorphic morphology of the virion in this realm.

## REALM *FLOREOVIRIA*, KINGDOM *SHOTOKUVIRAE*

Realm *Floreoviria* is the largest of the four realms formerly assigned to *Monodnaviria*, comprising three phyla, *Commensaviricota*, *Cossaviricota*, and *Cressdnaviricota*, which collectively include 29 families with 401 genera and 2,351 species for viruses with small, circular or linear ssDNA or dsDNA genomes (Fig. 1). Certain families in the phyla *Cossaviricota* (*Bidnaviridae* and *Parvoviridae*) and *Cressdnaviricota* (*Geminiviridae*, *Genomoviridae*, and *Nanoviridae*) harbor multipartite viruses, i.e., viruses with segmented ssDNA genomes that encapsidate each segment in a separate particle (29).

Floreovirians infect a wide range of eukaryotic hosts, from protists, to plants, to animals including humans. All members of this realm produce small icosahedral capsids, usually built based on the *T* = 1 lattice and less commonly on the *T* = 3 or *T* = 7 lattices (Fig. 1). The MCPs of the vast majority of characterized floreovirians have the SJR fold, but in some lineages, e.g., bacilladnavirids and an informal group known as “cruciviruses” (30), the ancestral SJR MCP has been replaced by a non-orthologous MCP, likely through recombination with distinct ssRNA viruses (31–35). In extreme examples, viruses classified within the same species encode unrelated MCPs (36). Furthermore, a recently described and currently unclassified cressdnaviricot associated with oomycete mitochondria apparently lacks the SJR MCP and instead encodes a membrane-embedded protein that was proposed to be involved in the formation of pleomorphic enveloped virus particles (37). Thus, unlike that of efunavirians, pleomovirians, and volverevirians, the morphogenetic module of floreovirians has undergone rampant diversification, involving multiple non-orthologous replacements.

Most floreovirians encode a relatively uniform replication module consisting of a fusion of the HUH superfamily endonuclease domain with a superfamily 3 helicase (S3H) domain. However, the replication module of floreovirians has undergone dramatic changes on at least three occasions (Fig. 1).

1. In members of the class *Papovaviricetes* (kingdom *Cossaviricota*), the HUH endonuclease domain is inactivated, whereas the catalytically active S3H domain is retained. The inactivated HUH domain of the replication proteins of papovaviricetes functions as an origin recognition domain rather than an endonuclease, and the replication does not proceed through an ssDNA intermediate (38, 39). Accordingly, papovaviricetes have circular dsDNA genomes rather than ssDNA genomes.
2. In members of the class *Mouviricetes* (kingdom *Cossaviricota*), the HUH domain of the RCRE was replaced by a protein-primed family B DNA polymerase, whereas the S3H domain of the ancestral HUH-S3H Rep was retained as the product of a separate gene (40).
3. The most radical change apparently occurred in the phylum *Commensaviricota* (family *Anelloviridae*). Its viruses retained the ancestral SJR MCP (41) but lost the RCRE (42). Recent preliminary evidence suggests that anellovirids do not encode any enzymes required for the replication of their genomes, which is performed entirely by the host DNA replisome (43).

These changes in the replication and morphogenetic modules notwithstanding, there is sufficient evidence supporting the common ancestry of all floreovirians. In particular, the unique two-domain organization of their replication protein has not been observed in Reps of known viruses outside of *Floreoviria* (Fig. 2). However, homologous Reps, with the same domain architecture, have been described in a particular group of bacterial plasmids (13). Thus, the ancestor of floreovirians is believed to have evolved from a distinct group of RCRE plasmids, independent of those of other viruses with small ssDNA or dsDNA genomes, justifying the recognition of *Shotokuvirae* as a separate realm.

To avoid potential confusions associated with the realm name *Monodnaviria* (as it wrongly implies *mono*phyly of ssDNA viruses and that all of them have ssDNA genomes), the realm *Monodnaviria* was renamed to *Floreoviria*.

Etymology: *Floreoviria*, after Latin “floreo” for blossom, bloom, flourish, referring to the bloom of diversity in the virus realm in terms of genome structures and topologies, mechanisms of replication, and virion structures.

## CONCLUDING REMARKS

The four kingdoms of the former realm *Monodnaviria* included four distinct assemblages of viruses that were found to be monophyletic within each kingdom but unrelated to viruses of the other kingdoms. This conclusion was reached through analysis of both the replication and morphogenetic modules, the two principal components of viruses with small genomes (10). Accordingly, to ensure the monophyly of the realms, the realm *Monodnaviria* was reorganized by moving three of the four kingdoms into separate new realms, *Efunaviria*, *Pleomoviria,* and *Volvereviria*, and renaming the realm *Monodnaviria* to *Floreoviria*. Originally, the former realm *Monodnaviria* was defined by the apparent conservation of the key replication enzyme, the HUH endonuclease involved in RCR initiation. However, a more detailed analysis of the fast-growing diversity of viruses with ssDNA genomes showed, first, that not all of them encoded an HUH endonuclease, and second, that some of the latter appeared to have originated independently, from different plasmids (10). For the updated taxonomy, a more nuanced approach was adopted, whereby the realms are brought together by the conservation of the morphogenetic module rather than the replication module, which varies within each of the four realms, even though the HUH RCRE remains the most common replicative enzyme. We cannot rule out that for one or more of the realms described here, evidence of independent origins will be obtained and lead to further splits. Nevertheless, the 4-realm taxonomy presented here, which is strictly based on the conservation of key genes, appears far more robust than the previous taxonomy that lumped all these viruses into *Monodnaviria*. The viruses with small DNA genomes comprising the four realms provide a striking example of viral genome plasticity and convergent evolution, whereby distinct groups of viruses originated independently but along similar paths through continuous recombination with plasmids and other viruses.
